# Misrepresented multiple endocrine neoplasia 2: Do the British Thyroid Association guidelines accurately predict thyroid cancer risk in high-risk groups with multiple endocrine neoplasia 2? A case series

**DOI:** 10.1177/1742271X241260225

**Published:** 2024-06-23

**Authors:** Arya Anthony Kamyab, Alex Weller, Kate Hulley, Gul Bano

**Affiliations:** St George’s University Hospitals NHS Foundation Trust, London, UK

**Keywords:** Medullary thyroid carcinoma, British Thyroid Association, multiple endocrine neoplasia

## Abstract

**Introduction:**

The incidence of thyroid nodules in the general population is around 40%. The British Thyroid Association U-grading has high sensitivity for identifying the common thyroid cancer subtypes (papillary and follicular). However, ultrasound features of the rarer medullary thyroid cancer differ, with lower sensitivity for ultrasound detection.

Hereditary medullary thyroid cancer accounts for 25% of cases, forming part of the multiple endocrine neoplasia syndromes (multiple endocrine neoplasia 2) and is associated with RET proto-oncogene mutation, for which gene testing is increasingly available. This study aims to evaluate British Thyroid Association U-grading for thyroid cancer risk stratification in this high-risk population.

**Case report:**

This was a retrospective review of four multiple endocrine neoplasia 2 patients referred for thyroid ultrasound. A total of 10 thyroid nodules were graded as part of routine evaluation, taken from an endocrine and genetics tertiary referral centre. Patients with identifiable RET mutation from March 2017 to February 2023 were reviewed.

**Discussion:**

Six patients had 10 thyroid nodules, of which 8 were graded as U2, 2 graded U3–5 and 8 confirmed as medullary thyroid cancer. However, two patients had no pathology data at the time of writing. For this cohort, U-grading and genetics were discordant, with RET gene testing more effective than ultrasound in cancer detection. All nodules should be considered high risk for medullary thyroid cancer, regardless of U-grade.

**Conclusion:**

Our data demonstrate that British Thyroid Association U-score has limited value for medullary thyroid cancer detection in this high-risk group and cannot be used for risk stratification or surveillance. As a rarer thyroid cancer subtype, medullary thyroid cancer and the high-risk multiple endocrine neoplasia 2 population are under-represented in British Thyroid Association 2014 guidance and deserve consideration in future editions.

## Introduction

The incidence of thyroid nodules in the general population is around 40%, increasing with age. The primary aim of ultrasound assessment is identifying malignancy.^1^ Despite being the commonest endocrine malignancy, thyroid cancer accounts for around 1% of all cancers, with annual UK incidence of 2–5 per 100,000 (compared with 40% for thyroid nodules).

With minor geographic variations, differentiated thyroid cancer subtypes (papillary thyroid carcinoma (PTC) and follicular thyroid carcinoma (FTC)) account for 90%–95% of new cases and are contributing to increasing thyroid cancer incidence globally.^1^ Medullary thyroid carcinoma (MTC) is rarer, representing 1%–5% of thyroid cancer cases, but up to 15% of thyroid cancer-related deaths. MTC has sporadic and familial forms with wide age range at onset, occurring typically around the fourth to fifth decades.^2^ Sporadic MTC accounts for around 80% of cases and is commonly unilateral with no endocrinopathies.^3^ Familial MTC (FMTC) accounts for 20%–25% of cases and arises either in the context of the multiple endocrine neoplasia 2 (MEN2) syndromes or as pure FMTC, for which genotype–phenotype correlations are increasingly established. The MEN2 syndromes are associated with RET proto-oncogene mutation and have predisposition for tumours involving two or more endocrine glands, including MTC for 95% of carriers.^4^ The RET proto-oncogene is also the major gene involved in Hirschsprung’s disease, with loss-of-function mutations identified in over 70% of cases and an association with long segments of aganglionic bowel. Sharing this susceptibility gene, RET-associated MTC has a widely reported association with personal or family history of Hirschsprung’s disease.^3^

An ultrasound scan (USS) is an extremely sensitive and non-invasive technique for identifying and risk stratifying thyroid nodules, while guiding targeted fine needle aspiration for cytological analysis (FNAC).^5^ In thyroid nodule ultrasound characterisation, several groups have devised grading systems combining multiple features to optimise diagnostic performance and triage which nodules require further FNAC evaluation, ideally safely detecting all thyroid cancers while minimising the number sampled.^6^ Among these include the Thyroid Imaging Reporting and Data System (TI-RADS) and British Thyroid Association (BTA) U-classifications. Under both classifications, the objective is to identify the less frequent malignant nodules from their more common benign counterparts, for which the features defined as conferring malignancy risk are derived from the more common differentiated cancers (PTC and FTC), for which sensitivity is extremely high (1/894).^7^ Under the BTA 2014 guidelines, benign features (U1–2) are regarded as not requiring FNAC unless the patient is at a high baseline risk of malignancy. Notably however, the pre-test clinical risk factors for malignancy do not include the MEN2 syndromes.^5^

For patients with known hereditary MTC, genetic risk stratification with RET testing is increasingly available, to determine the timing of prophylactic thyroidectomy.^3^ Approximately, 85% of mutations responsible for FMTC are now known and genetic testing detects nearly 100% of these. In the familial syndromes, specific RET proto-oncogene mutations are associated with disease aggressiveness and prognosis, such that prophylactic thyroidectomy is recommended by the age of 10 for all recognised RET-oncogene mutation carriers.^3,8^ However, ultrasound still plays a key role in diagnosing de-novo hereditary cases and sporadic MTC.

As genetic testing is a relatively new technique, hereditary RET-oncogene mutation MTCs are sometimes detected de-novo with no family history, while somatic RET point mutations have also been identified in up to 50% of patients with sporadic MTC.^3^ For de-novo cases, ultrasound is often the initial diagnostic test, meaning maximising sensitivity for MTC is imperative in identifying cancer in this high-risk group.

The aim of this report was to highlight the limitations of applying BTA classification in the context of hereditary MTC, by looking at a series of patients in whom histologically confirmed MTC was initially reported at ultrasound as possessing benign or indeterminate features (U2–3).

## Case series

This was a retrospective review of patients with MEN2 referred for thyroid ultrasound as part of routine clinical risk stratification. Nodule grading was performed prospectively by the sonologist at the time of scanning. The scans were not read again retrospectively to reflect clinical practice in a real-world thyroid service. If there was discrepancy between the frontline scan and the Multidisciplinary team (MDT) grading, the latter was chosen as correct, and feedback offered to the sonologist. All graded nodules were subsequently confirmed as MTC, despite sonographic U2–3 classification. The cases were identified from a population of individuals with MEN syndromes at a single endocrine and genetics tertiary referral centre. Ethics approval and patient consent were not needed due to the study’s retrospective nature and use of clinically routine data. This included all new patients with an identifiable RET mutation between March 2017 and February 2023.

## Case 1

**Figure 1.**
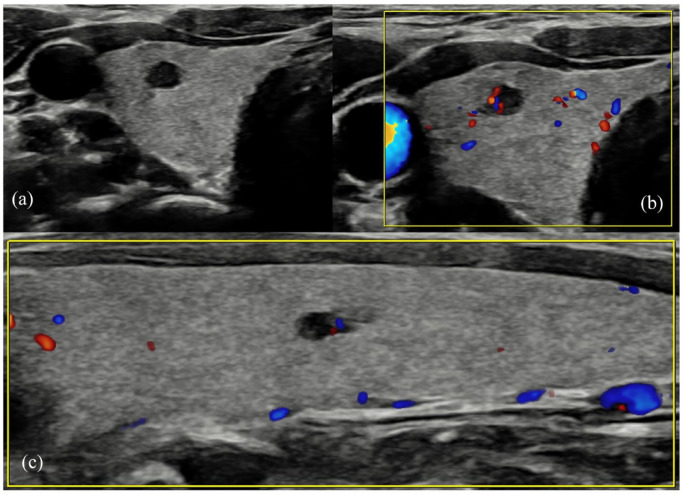
(a) Right thyroid well-defined hypoechoic 3-mm nodule, easily mistaken for an anechoic cyst nodule, especially given the small size – graded U2 at the time of scanning. (b) The same nodule demonstrating peripheral vascularity only. (c) A further 3-mm nodule in the same patient in the left lobe of the thyroid, demonstrating the same imaging characteristics. The nodules were both histologically confirmed as medullary thyroid carcinoma.

An 18-year-old female was referred following routine blood testing by her general practitioner, for endocrinology assessment for primary hyperparathyroidism following worsening hypercalcaemia and hypophosphataemia, despite good vitamin D levels. A sestamibi (MIBI) scan was suspicious of a right-sided parathyroid adenoma, while genetic testing confirmed heterozygosity for a pathogenic RET variant, conferring risk of developing other MEN2-related tumours. Thyroid ultrasound revealed normal thyroid echotexture, within which a solitary, well-defined, near anechoic 4-mm nodule was graded as U2, while a juxta-thyroid, well-defined, hypoechoic nodule adjacent to right thyroid lower pole corresponded with MIBI tracer uptake, in keeping with a parathyroid adenoma (Figure 1).

Genetics data were unavailable at the time of the initial USS and MEN2 status was confirmed prior to MDT discussion. Performed due to this genetic risk reported at MDT, subsequent FNAC of the right thyroid 4-mm nodule confirmed MTC (Thy5) and the patient proceeded to total thyroidectomy with level VI clearance and removal of the parathyroid adenoma.

## Case 2

A 38-year-old (AC) was referred for genetic screening with clinical left neck swelling, after a first cousin was found to have MEN2A following a de-novo diagnosis of thyroid MTC. AC had been diagnosed with Hirschsprung’s disease after birth and was found to carry a pathogenic RET variant consistent with MEN2A. These genetics data were available in the clinical information at the time of both initial USS and the subsequent MDT discussion.

**Figure 2.**
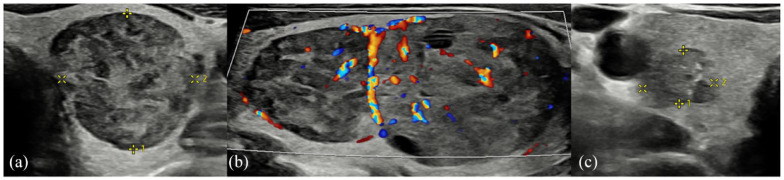
(a) Left thyroid well-defined ovoid 2-cm nodule with mildly hypoechoic (cf. background thyroid) heterogeneous echotexture. (b) Images of the same nodule in longitudinal section demonstrates two closely approximated nodules with mixed vascularity. Cystic spaces are also demonstrated within the larger of the two nodules. These were graded as U3 at the time of scanning. (c) Within right thyroid, an ill-defined 8-mm, mildly hypoechoic nodule with internal echogenic foci (representing probable microcalcification) was graded as U5.

Within the left thyroid at ultrasound, two confluent mildly hypoechoic nodules (cf. strap muscles) had moderate internal vascularity and occasional cystic components, classified as U3, while within right thyroid, a hypoechoic lobulated 8-mm nodule with macrocalcification was graded U5 (Figure 2). FNAC confirmed bilateral Thy5 MTC, for which total thyroidectomy with level VI clearance was performed.

## Case 3

A 63-year-old (BD) man referred to genetics after a diagnosis of MEN2A in his daughter and nephew, each sharing the same RET mutation, making BD an obligate carrier as confirmed at genetic testing. At endocrinology referral, his calcitonin level was elevated (129 ng/L) and apart from thyroid nodules, computed tomography chest, abdomen and pelvis were normal.

**Figure 3.**
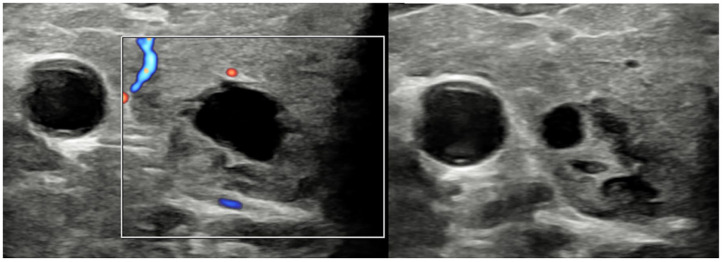
Both thyroid lobes contain multiple well-defined, mixed cystic and solid nodules with no internal Doppler vascularity. These were described as cystic or spongiform – U2 nodules initial visit.

Thyroid ultrasound showed bilateral well-defined, ovoid, mixed cystic and isoechoic solid nodules measuring up to 16 mm, all graded U2 (Figure 3). However, triggered by increased DOTATATE uptake corresponding with the largest 16-mm right-thyroid nodule, FNAC confirmed Thy5 MTC, necessitating total thyroidectomy and bilateral level-VI dissection. While genetic diagnosis was unavailable at the time of ultrasound, a family history of MEN2 was recorded. Both MEN2 status and FNAC were available at the time of MDT discussion.

## Case 4

A 32-year-old lady (CE) with neonatal history of Hirschsprung’s disease was referred for genetic screening following RET oncogene mutation diagnosis in her mother, a mutation that was confirmed for CE, in keeping with a diagnosis of MEN2A.

**Figure 4.**
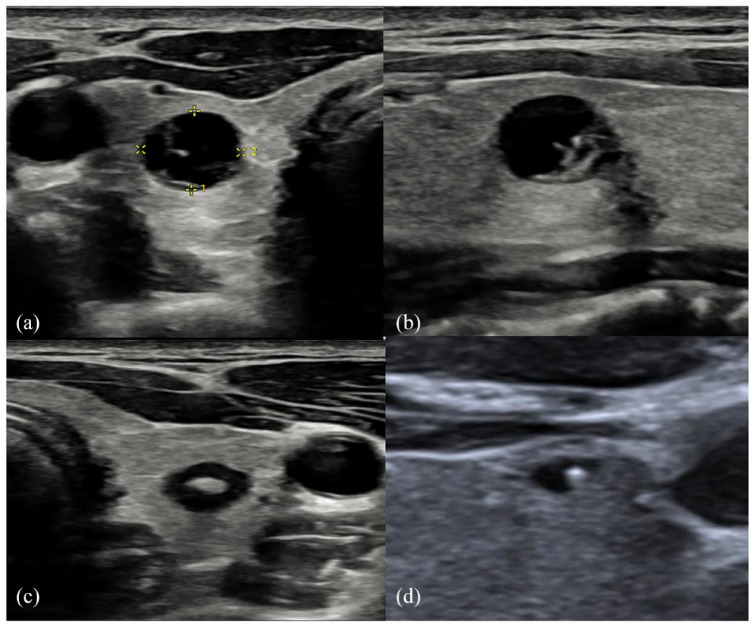
(a) Right thyroid well-defined cystic appearing 8-mm nodule with posterior acoustic enhancement. (b) The same right thyroid nodule or cyst demonstrating internal echogenic focus, initially misinterpreted as colloid (as in Figure 4(d)) but revised at Multidisciplinary Team (MDT) to dystrophic calcification due to posterior shadowing. (c) A contralateral left thyroid nodule (demonstrating minimal peripheral Doppler vascularity). However, subtle posterior acoustic shadow is demonstrated from a central echogenic focus, also in keeping with calcification rather than colloid. The nodules were all described as U2 on the initial study; however, they were confirmed histologically as medullary thyroid carcinoma. (d) Transverse image of a right thyroid lobe in another (low-risk) patient for comparison, showing an anechoic cyst with central echogenic focus that demonstrates colloid ‘ring-down’ artefact, in keeping with colloid cyst.

On endocrinology referral, elevated serum calcitonin to 23.8 ng/L was identified, while thyroid ultrasound showed a homogeneous background parenchyma, with bilateral well-defined, near anechoic 7–8-mm nodules with posterior enhancement and no discernible Doppler vascularity. One of the left-sided nodules had internal echogenicity with posterior shadow that was initially interpreted as colloid (hence U2 bilaterally; Figure 4). The MEN2A status was recorded in the clinical information at the time of the initial ultrasound. A DOTATATE Positron Emission Tomography (PET) scan did not reveal any DOTATATE avid lesions above background thyroid uptake.

However, at MDT review, interpretation on the left was revised to coarse or dystrophic calcification within a U3 nodule. FNAC confirmed Thy5 MTC within both lobes and was followed by total thyroidectomy with selective neck dissection.

Two further patients with MEN2 had 2–5 mm nodules described as U2 at ultrasound, but pathology data were not available at the time of writing. With incomplete information, these patients were excluded from this series.

## Discussion

As this group of MEN2 patients have a high baseline risk of MTC, population-based thyroid ultrasound classification systems misrepresented nodules as having either benign or indeterminate features, but with subsequent confirmation as MTC. There have been no studies validating BTA or TI-RADS grading systems in this population, for whom our results confirm limited sensitivity for malignant nodule detection. From this, we hypothesise that benign classification under BTA (and by extrapolation TI-RADS) in this context does not outweigh the MTC risk determined from genetic RET mutation testing and should not be misinterpreted as confirming benignancy. For this group, in whom lifetime MTC risk approaches 100%, the applicability of BTA and TI-RADS grading as decision-making tools is questionable. Staging imaging and thyroidectomy are warranted in all RET mutation-positive patients in the MEN population, regardless of ultrasound U-grade.

The now widely adopted thyroid nodule ultrasound grading systems include features based on background population risk, resulting in representation from the commoner cancer subtypes (PTC > FTC), relatively lower representation for MTC and even lower representation of the rare MEN2 syndromes. In the low-risk population from which the grading systems were constructed, a high sensitivity for cancer detection has been achieved; in one UK study applying BTA U-classification to 1225 nodules in 964 patients, of the 894 nodules identified as benign on ultrasound (U2), only one cancer was discovered.^7^ However, prior to adoption of the TI-RADS and BTA systems from 2014 onwards, MTC had been recognised as having sonographic features that are distinct from differentiated carcinomas.^9^ In one cohort, MTCs less frequently possessed ‘suspicious’ ultrasound features compared with PTCs,^10^ while in another, histologically confirmed MTC was more frequently misdiagnosed as benign at ultrasound than PTC.^9^

From these studies, compared with PTC, MTC is sonographically less likely to have irregular borders, microcalcifications or a taller than wide shape (all ‘suspicious’ features for differentiated thyroid carcinoma), while having a higher frequency of ‘reassuring’ features such as cystic change, homogeneous echotexture, circumscribed margin and oval shape. In addition, colloid-related comet tail reverberation artefact on ultrasound is a common benign feature on ultrasound, especially in the context of cystic nodules. This reverberation is however also seen in dystrophic calcification of MTC (as in case 4), leading to potential benign misinterpretation. These observations demonstrate a tendency towards lower ultrasound sensitivity for MTC than for PTC, contributing to potential false benign characterisation.^9^ In the current ultrasound classification systems, where cancer detection must be balanced against anxiety and morbidity of over-investigation, some features that are common in the rarer MTC are not included as suspicious for malignancy. This is likely due to the under-representation of MTC in data from which the guidelines were constructed and raises questions about which nodules to sample to avoid missing MTC. In addition, for patients with confirmed MTC (both sporadic and familial), the role of ultrasound in risk stratification of other nodules (including in the contralateral lobe) and in screening first-degree relatives requires further evaluation. For these higher risk groups, U-grading (and TI-RADS) nodule classification is not validated in the literature to date, warranting consideration in future guidance.

For three of four patients in our series, despite the ultrasound referral recording either a genetic predisposition or family history of MTC, initial radiological risk stratification was based on U-grading. Applicability of this grading was first questioned at MDT. This emphasises the need for greater sonographer awareness of the limitations of U-grading in high-risk groups. However, any proposed amendment to increase U-grading sensitivity for MTC in this rare MEN2 subgroup would be at the expense of diminished specificity for the wider population and increase in invasive interventions. To avoid this, pre-ultrasound identification is ideal, through comprehensive clinical evaluation prior to referral, as well as MDT review as soon as the familial risk is known.

The main limitation of case series such as this is sample size and future studies evaluating the risk of MTC in the MEN2 population in comparison to the background population presenting with U2 thyroid nodules are needed to validify the hypothesis-generating findings in this case series.

## Conclusions

1. Under currently adopted ultrasound classification systems, sensitivity for MTC is potentially lower than for PTC and future iterations of TI-RADS and BTA classification systems should include specific mention of MEN syndromes and FMTC.
2. Clinical assessment of hereditary thyroid cancer syndromes should be a part of the first clinical episode in a patient with malignant thyroid disease.
3. In patients with known hereditable genetic cancer syndromes associated with MTC (RET oncogene mutation positive), this information must be specified in the referring history for the ultrasound examination in all cases and benign classification at ultrasound should not defer FNAC or thyroidectomy.
4. For non-heritable confirmed MTC, ultrasound screening of first-degree relatives may be considered, although data supporting diagnostic accuracy of TI-RADS and BTA U-classification is lacking and a low threshold for fine needle aspiration may be considered, alongside biochemical risk stratification.
5. Ultrasound-based screening for thyroid disease should be a part of long-term surveillance in patients with multi-tumour genetic syndromes prone to developing thyroid cancer.
